# Testing a Culturally Tailored Advance Care Planning Intervention (MY WAY) for an American Indian Tribe: Protocol for a Quasi-Experimental Waitlist Control Design

**DOI:** 10.2196/50654

**Published:** 2023-12-29

**Authors:** Elizabeth Anderson, R Turner Goins, Emily A Haozous, April Schweinhart

**Affiliations:** 1 Pacific Institute for Research and Evaluation Chapel Hill, NC United States; 2 College of Health and Human Sciences Western Carolina University Cullowhee, NC United States; 3 Pacific Institute for Research and Evaluation Albuquerque, NM United States; 4 Pacific Institute for Research and Evaluatoin Louisville, KY United States

**Keywords:** American Indian or Alaskan Native, advance care planning, end-of-life care

## Abstract

**Background:**

American Indian and Alaska Native peoples experience poor end-of-life care, including more hospitalizations and lower use of hospice and do-not-resuscitate orders. Although advance care planning (ACP) can improve end-of-life care, ACP rates are disproportionately low in American Indians and Alaska Natives.

**Objective:**

We culturally tailored and delivered an existing evidence-based ACP program for an American Indian tribal community. Here, we present the protocol for assessing the intervention’s feasibility and efficacy.

**Methods:**

We measured feasibility via participant recruitment, participants’ evaluation (acceptability, appropriateness, comprehension, and satisfaction), and intervention fidelity. Recruitment was measured with participant screening, eligibility, enrollment, and retention. Participant’s evaluation of the intervention was measured with surveys. Fidelity was measured with direct observation and the Make Your Wishes About You (MY WAY) Fidelity Checklist Tool. To assess the intervention’s efficacy, we used a quasi-experimental waitlist control design with 2 cohorts who were surveyed each on three separate occasions. The intervention’s efficacy was assessed by the following: ACP barriers and facilitators as well as ACP self-efficacy, readiness, and completion.

**Results:**

A total of 166 participants were screened for eligibility; 11 were deemed ineligible, and 155 participants were enrolled in the study. Of those enrolled, 113 completed the intervention and will be included in subsequent analyses. We finalized data collection in January 2023, and analyses are underway. Study enrollment was successful, and we expect that participants will report high levels of acceptability, appropriateness, comprehension, and satisfaction with the intervention. We expect that the intervention was implemented with fidelity and will demonstrate decreases in ACP barriers and increases in ACP facilitators, self-efficacy, readiness, and completion.

**Conclusions:**

Enrolling over twice as many participants as we had hoped suggests that members of this tribal community are willing to engage in end-of-life ACP. We were able to implement a waitlist study design to show that a culturally tailored ACP program for a tribal community is feasible.

**Trial Registration:**

ClinicalTrials.gov NCT05304117; https://clinicaltrials.gov/study/NCT05304117

**International Registered Report Identifier (IRRID):**

DERR1-10.2196/50654

## Introduction

### Background

American Indian and Alaska Native peoples experience poor end-of-life care, including a higher likelihood of being hospitalized at the end of life; receiving futile treatment at the end of life; and lower rates of hospice [1], palliative care, do-not-resuscitate orders [2-4], and advance care plans [3,5]. Advance care planning (ACP) is a process that allows people to make health care decisions if they are unable to speak for themselves because of serious or terminal illness. ACP is associated with goal-concordant care, decreased anxiety about end-of-life decision-making, and reduced stress among primary caregivers [6].

ACP rates are disproportionately low among American Indians and Alaska Natives. Research with 270 American Indian and Alaska Natives in the Northwest Plains found that none had heard of palliative care and 93% had never heard of a living will [4]. When compared to non-Hispanic White people, American Indians and Alaska Natives were half as likely to have a living will or a health care power of attorney [3]. A medical chart review of a New Mexico Indian Health Service facility found that information about advance directives, as required by the Patient Self-Determination Act, was in only 20% of charts [7]. Despite being twice as likely to die from type 2 diabetes and 4 times as likely to die from chronic liver disease than non-Hispanic White people [8], little work has been done to increase opportunities for American Indians and Alaska Natives to express their end-of-life care wishes. To date, there are no randomized controlled trials (RCTs) that have demonstrated the efficacy of ACP for American Indians and Alaska Natives [5], and there are limited other ACP research studies with American Indians and Alaska Natives [2,3,9-11].

The lack of ACP among American Indians and Alaska Natives is not a reflection of unwillingness to engage in conversations about death [4,9,12]. Rather, American Indians and Alaska Natives are presented with formidable barriers to ACP including lack of access to such opportunities and experiences of health care professionals’ stereotypes that American Indians and Alaska Natives do not want to engage in end-of-life conversations [13]. Further, typical ACP models may be unsuitable for many American Indians and Alaska Natives because they are conventionally delivered by health care professionals in clinical settings that American Indians and Alaska Natives have historically valid reasons for not trusting [11,13-16]. ACP approaches for American Indians and Alaska Natives should be developed with guidance and input from the intended community [11,17] and incorporate culturally relevant values [18] as well as beliefs and practices concerning the end of life and death, which vary within and across Indigenous populations [19].

A previous RCT study demonstrated that the program, Make Your Wishes About You (MY WAY), increases ACP self-efficacy, readiness, and completion rates for patients with chronic kidney disease [20,21]. However, as the Consolidated Framework for Implementation Research asserts, interventions that are not tailored to a population of interest are usually a poor fit [22]. To deliver interventions that are sensitive to a group’s worldview, language, cultural meanings, and values, a systematic approach to tailoring is suggested [23,24]. Tailoring includes modifying the intervention delivery, that is, the delivery person, channel, or location, and modifying content, such as certain information in the intervention materials [25].

### Objectives

The objective of our study was to use a community-based participatory research (CBPR) approach to culturally tailor the existing MY WAY [21] for a tribal community. Specifically, our study had three aims: (1) culturally tailor MY WAY for a specific tribe, (2) assess the feasibility of the culturally tailored MY WAY, and (3) examine the efficacy of the culturally tailored MY WAY. At the successful completion of this project, we expect to develop a feasible, culturally tailored MY WAY that, through a quasi-experimental waitlist control design, shows promise as an efficacious program. Here, we describe the protocol for assessing the feasibility and efficacy of the culturally tailored MY WAY.

## Methods

### Ethical Considerations

The project received approval from our institutional review board (blinded to protect confidentiality; #2022-01-24-01-02), a tribal institutional review board, and a tribal council (resolution #412-2021).

### Conceptual Framework

Our approach relied on the Consolidated Framework for Implementation Research, which provides a practical structure for complex, interacting, multilevel, and momentary constructs by consolidating key elements from implementation theories [22]. The framework’s main premise is that for implementation to be effective, the following domains must be considered: intervention characteristics, setting, individuals involved, and implementation process. Methodologically, our approach also adhered to the CBPR principles, including recognizing the community as a unit, building on strengths and resources within the community, facilitating collaborative partnerships in all phases of the research, integrating knowledge and action for the mutual benefit of all partners, promoting a co-learning and empowering process that attends to social inequalities, involving a cyclical and iterative process, and disseminating knowledge gained to all parties [2,26].

### Setting

Our study was conducted with a federally recognized tribe whose lands span five rural counties with approximately 16,000 enrolled members (personal communication with the personnel at the tribal enrollment office, May 23, 2023; the name of person and tribe deidentified for confidentiality).

### Intervention Characteristics

#### Overview

**Figure 1:**
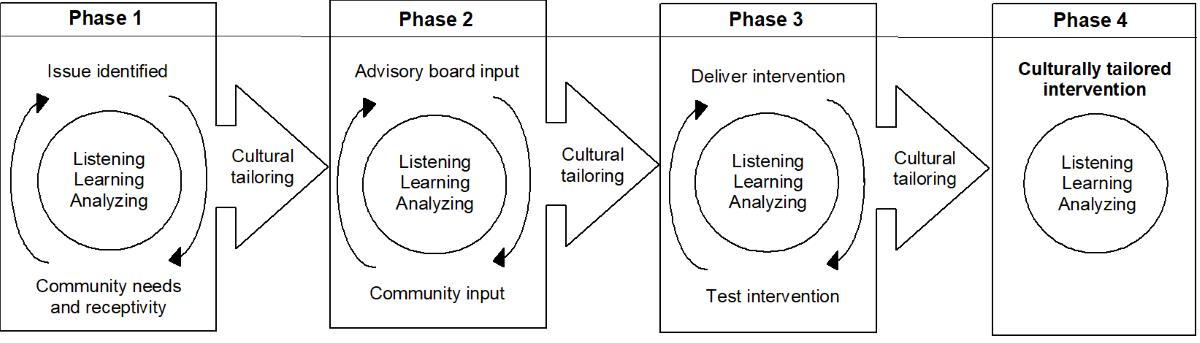
Cultural tailoring framework.

The original MY WAY project developed and used *Planning Today for Tomorrow’s Healthcare*, an ACP patient guide, and *A Curriculum Guide for ACP* to assist health care providers in having ACP discussions with patients in a nephrology clinic. Both guides are grounded in the stages of change theory and use motivational interviewing techniques [21]. The previous RCT demonstrated that the MY WAY curriculum was effective in increasing self-efficacy, readiness, and completion of ACPs when used with a trained coach, especially in ACP “conversation ready” health care settings [27]. For the cultural tailoring effort, we were guided by the Consolidated Framework for Implementation Research and were informed by community engagement. Our approach included a cyclical process of listening, learning, and analyzing at each phase. The framework is shown in Figure 1 and described below.

In phase 1 of the cultural tailoring process, we identified community needs and receptivity through collaboration with local leaders and health care providers, and by holding two listening sessions. This phase also included tailoring the delivery of the program to create a 2-step intervention: (1) community information sessions and (2) one-on-one sharing sessions with project staff (support stars) who were trained to deliver the intervention. At the end of this phase, we applied for and received National Institutes of Health funding in response to PAR 19-0587 entitled “Strategies to Provide Culturally Tailored Palliative and End-of-Life Care for Seriously Ill American Indian and Alaska Native Individuals.”

Phase 2 included the greatest number of activities. First, we created a community advisory board (CAB) and a professional advisory board (PAB). In CAB and PAB meetings, we shared data from the listening sessions and discussed ways to incorporate the feedback. We also hosted four additional listening sessions to obtain feedback on the original MY WAY materials. We used a grounded theory approach to analyze the listening session data to guide subsequent tailoring. We held an additional meeting with the CAB and PAB to share findings and elicit feedback that was used to further tailor MY WAY.

After the first revisions of MY WAY materials, we met individually with 7 of the listening session participants to solicit their reactions to the materials’ usability and acceptability. We then met the CAB and PAB for a third time to share the interview findings and incorporate additional revisions to further tailor MY WAY. In phase 2, we also used the process, as described by Lynn [28], to quantitatively evaluate the content validity of the culturally tailored materials. A self-administered web-based survey was sent to all CAB and PAB members that included items about the personal and curriculum guides concerning the appropriateness of the images and language, and if the content was culturally relevant, culturally sensitive, respectful, and clear. If a respondent indicated an issue, they were then prompted for suggestions as to how to address it.

Phase 3 included implementing, delivering, and testing the culturally tailored MY WAY. First, participants attended a community information session on ACP, which was held at a venue within the tribal community. After attending a community information session, participants scheduled a one-on-one meeting or a sharing session. Topics covered in the community information session and sharing session included an overview of the project, health care agents, future health care wishes, writing an ACP, sharing the ACP, and Physician Orders for Life Sustaining Treatment (POLST) forms. The sharing sessions allowed participants to ask personal and private questions of the support star. These sessions were held in a location of the participant’s choice (eg, their home or community club), and they were encouraged to bring family members. The support star also notarized ACPs, if desired, at any point in the process. The implementation phase lasted approximately 10 months. Finally, in phase 4, the investigators met again to conduct a final tailoring of the materials based on impromptu feedback that was received during implementation (phase 3). This included eliminating the term “healthcare agent” and adding information on how participants can share their notarized ACP with their health care provider. More information on the cultural tailoring can be found elsewhere (Goins, Haozous, Anderson, and Winchester, unpublished data, under review).

### Research Design

#### Feasibility

Assessing feasibility was a primary objective for this study as there are no clinical trials on ACP interventions for American Indians and Alaska Natives and limited other work for ACP with this population. Although American Indians and Alaska Natives are willing to talk about and plan for end-of-life events, it was still necessary to assess whether there were other reasons that American Indians and Alaska Natives have lower than usual ACP rates. Conceptually, feasibility was measured with participant recruitment, participant evaluation (acceptability, appropriateness, comprehension, and satisfaction), and the fidelity of the intervention.

#### Efficacy

In addition to determining feasibility, we also sought to measure the efficacy of the culturally tailored intervention to address the disproportionate rates of ACP for this population. We used a quasi-experimental waitlist control study design to test the hypothesis that the intervention participants would experience decreased ACP barriers and increased ACP facilitators, self-efficacy, readiness, and completion rates. Rather than deny a portion of our sample the intervention, as in a traditional RCT, we planned to implement both steps of the intervention twice such that our intervention group, cohort 1, would comprise those who participated in the first round of both the community information sessions and sharing sessions. Our comparison group, cohort 2, would comprise those who participated in the second round of both the community information sessions and sharing sessions. To test the efficacy, we used the combined 2-cohort sample of those who participated in both steps of the intervention and compared intervention baseline measures to those measured post program.

### Study Population and Sample Size

Eligibility criteria for the study population included tribal members, spouses of tribal members, tribal first descendants, and other American Indians and Alaska Natives residing in the tribal community, aged ≥18 years, and residing within the tribe’s service area. Participants with cognitive impairments were excluded. Participants were recruited via purposive sampling. Existing collaborative relationships established in the tailoring phase helped the recruitment process. Recruitment efforts included numerous in-person announcements at tribal venues at events, a video on the local television station, social media postings, and several billboards.

Based on the risk ratio of completing an ACP indicated in the previously implemented MY WAY intervention (1.79, 95% CI 1.18-2.72) and using 90% power as a threshold, we used G*Power (Axel Buchner) with 3 predictors and the primary outcome of ACP completion to estimate that we would need at least 60 participants to find effects. As effect sizes for ACP self-efficacy and readiness were smaller in the original intervention, our goal was to enroll a total of 70 participants with 35 in each cohort.

### Procedure

#### Overview

##### Feasibility

Recruitment was measured by documenting the number of potential participants in attendance and completing each intervention event or measure. Participant’s evaluation of the intervention was measured twice: once at the end of the community information session with a self-administered survey and once 1 week after the sharing session with a telephone interviewer-administered survey. We conducted fidelity checks on 40% of the community information sessions and 18% of the sharing sessions.

##### Efficacy

Procedures varied across cohorts. Cohort 1 completed intervention baseline surveys immediately before the intervention and completed postprogram surveys about 9 weeks after the intervention. Cohort 1 also completed follow-up surveys approximately 6 months after their intervention baseline surveys. Cohort 2 completed a control baseline survey approximately 4 months before the intervention, and a second intervention baseline survey right before the second set of community information sessions. Cohort 2 then completed a postprogram survey approximately 3 months later. Table 1 describes the data collection schedule by cohort.

**Figure 2:**
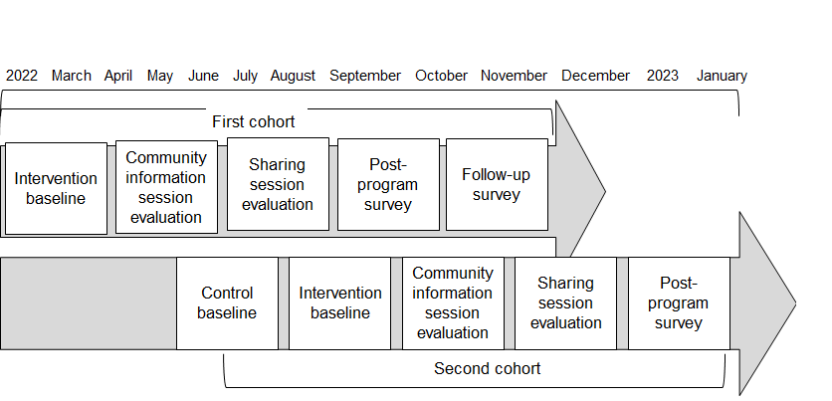
Cohorts' timeline.

A timeline describing the steps of the intervention and data collection is shown in Figure 2.

**Table 1:** Data collection schedule by cohort.

Data collection				Timeframe		Format
**Cohort 1**						
	Intervention baseline		≤1 week before CIS^a^		Interviewer-administered in person	
	**Community information session**					
		CIS evaluation	Immediately after on site		Self-administered paper/pencil	
	**Sharing session**					
		SS^b^ evaluation	1 week post SS		Interviewer-administered telephone call	
		Postprogram survey	9 weeks post CIS		Interviewer-administered telephone call	
		Follow-up survey	6 months post CIS		Interviewer-administered telephone call	
**Cohort 2**						
	Control baseline		4 months before CIS		Interviewer-administered in person	
	Intervention baseline		≤1 week before CIS		Interviewer-administered in person or telephone call	
	**Community information session**					
		CIS evaluation	Immediately after on site		Self-administered paper/pencil	
	**Sharing session**					
		SS evaluation	1 week post SS		Interviewer-administered telephone call	
		Postprogram survey	12 weeks post CIS		Interviewer-administered telephone call	

^a^CIS: community information session.

^b^SS: sharing session.

### Measurement

#### Feasibility Measurements

##### Recruitment

Recruitment was captured by documenting the number of potential participants who were screened, deemed eligible, enrolled, and engaged in the full intervention, and the retention rates (number who completed the entire intervention, number who completed postprogram surveys from both cohorts, number in cohort 1 who completed the follow-up survey, and the number who dropped out from the control baseline to the intervention baseline in cohort 2).

##### Participant Evaluation

Acceptability, appropriateness, comprehension, and satisfaction of the intervention experience were assessed with a self-administered survey immediately following the community information sessions. Sharing session evaluations measuring the same concepts were administered by interviewers via telephone. Acceptability and appropriateness of the community information sessions and the sharing sessions were captured with the Acceptability of Intervention Measure and the Intervention Appropriateness Measure [29]. Comprehension and satisfaction were assessed with eight items, with response options ranging from strongly agree to strongly disagree, and a single item about how they rated the session, with response options ranging from excellent to poor.

#### Implementation Fidelity

Implementation fidelity was measured with the MY WAY Fidelity Checklist, which was developed and used in the MY WAY RCT. This checklist tool was a modified version of the Treatment Adherence Fidelity Tool [30], which allowed us to assess the adherence to the intervention delivery and content, intervention pacing, participant responsiveness, intervention delivery quality index, and support stars’ competence level in judgment and delivery.

#### Efficacy Measurements

##### Barriers and Facilitators

We also used the ACP Facilitators and Barriers survey instrument [31] used in the original MY WAY RCT [27]. Barriers and facilitators were created based on findings from a former study [31] that found barriers to be related to perceptions of relevancy, personal barriers, relationship concerns, information needs, time constraints, and problems with the advance directives.

##### Self-Efficacy and Readiness

We used the 9-item ACP engagement survey instrument, including measures of self-efficacy and readiness as identified in behavior change theory [32,33]. Self-efficacy and readiness items have Likert responses of “not at all, a little, somewhat, fairly, extremely” and have good reliability and discriminant validity [32,33].

##### ACP Completion

ACP completion at baseline was operationalized with the number of participants who answered yes to the following question on the baseline survey (intervention and control): “Do you have an advance care plan?” The project coordinator confirmed a survey self-report of ACP completion by reviewing documentation as to whether the support star had notarized the advance care plan, which was confirmed with the official notary records.

### Analyses

At the time of this publication, analyses for this study were not complete. Below, we report on our planned analysis.

#### Feasibility

Recruitment will be reported in raw counts. Participant evaluation and fidelity will be analyzed using descriptive statistics (mean, SD, and frequency scores for each).

#### Efficacy

Participant demographic characteristics will be examined using descriptive statistics and stratified analyses (eg, by demographics, attendance, and ACP completion) to examine the association between demographics and intervention outcomes. Since there may likely be significant attrition from community information sessions to sharing sessions, we will also run Heckman selectivity analyses to determine if there were differences between those who completed both intervention steps and those who completed only baseline measures for community information sessions. If there are differences in attrition, we plan to include the inverse Mills ratio. The comparison of measures between each cohort’s intervention baseline scores will ensure that there are no significant differences between the cohorts that would keep them from being combined. ACP barriers, facilitators, self-efficacy, readiness, and ACP completion will then be compared between those who do and do not engage in both intervention steps and between cohorts 1 and 2. The comparison analysis will consist of Student *t* tests or a similar nonparametric test for differences in means if necessary.

After these preliminary analyses, the cohorts will be combined to examine changes in ACP outcomes (barriers, facilitators, self-efficacy, readiness, and completion) from the intervention baseline to the postprogram survey results. In the full sample of participants who experienced both steps of the intervention, the intervention baseline results for cohort 1 will be combined with the intervention baseline results for cohort 2 (as both occurred immediately before the intervention began) and compared to the combined sample of the postprogram surveys. Repeated measures of ANOVA will be used to compare these outcomes before and after the intervention. We will also assess changes in cohort 1’s ACP outcomes from the intervention baseline to the postprogram survey and compare them to changes in cohort 2’s ACP outcomes from the control baseline to the intervention baseline. Cohort 2 received no intervention between the control baseline and the intervention baseline, making this comparison analysis a control for natural variations over time. To ensure that changes can be attributed to the intervention and not the result of the passage of time or contamination from participants hearing about ACP in the community, we will measure two changes: (1) the change in cohort 1’s intervention baseline to postprogram scores and (2) cohort 2’s change from the control baseline to the intervention baseline.

## Results

This project was funded in March 2021 and the cultural tailoring process began immediately. The cultural tailoring of the delivery and content of MY WAY has been completed and the intervention has been delivered to both cohorts. Data collection for the intervention began in February 2022 and ended in January 2023. A total of 166 participants were screened for eligibility, and we enrolled 155 participants in the study, including 114 participants in cohort 1 and 41 in cohort 2. A total of 113 participants (cohort 1: n=81; cohort 2: n=32) completed both steps of the intervention and data collection. Data analyses of the intervention’s feasibility and efficacy have begun and are expected to be completed in the fall of 2023. During the project, we have kept our study participants informed through a periodic project newsletter. The culturally tailored MY WAY curriculum and personal guides are available for free on the project website.

## Discussion

### Principal Findings

There is a dearth of research on ACP interventions with American Indians and Alaska Natives and no culturally tailored ACP interventions for American Indians and Alaska Natives, despite having a high occurrence of chronic diseases such as diabetes [8], high rates of preventable hospitalizations [34], high premature mortality rates [35], and low hospice use rates compared to White people [2,36]. This study culturally tailored an existing evidence-based ACP program for an American Indian tribe. Though our data analyses have not been finalized, we were able to enroll over twice the number of participants (n=155) as hypothesized (n=70). Further, we successfully implemented a waitlist control design with this population.

### Interpretations, Implications, and Comparisons

Our study’s success at enrolling 155 participants and completing the waitlist design suggests that members of this tribal community were willing to engage in end-of-life discussions and ACP. Prior studies have indicated that providers often believe American Indians and Alaska Natives are reluctant to engage in end-of-life care or that discussions about the end of life are taboo [11-14]. We hypothesize that this study will show that tailored ACP interventions can increase facilitators; decrease barriers; and demonstrate increases in self-efficacy, readiness, and completion rates [4,12]. Tailoring interventions is believed to generate greater buy-in, acceptance, and ultimately the intended outcomes, and is considered preferable when working with populations in which the evidence-based program was not originally tested.

### Limitations

This study has several limitations, most specifically in that it only represents 1 of 574 federally recognized tribes [37], each with its own set of values and preferences concerning the end of life. However, the process of culturally tailoring and engaging the community can be replicated so that other tribal nations and their members can benefit from ACP. Like other CBPR studies, our approach required flexibility with the community’s needs, which created challenges in adhering to the strict timeline for the RCT. Instead, we adopted the waitlist approach. Additional cultural tailoring and testing of the MY WAY ACP intervention will be needed to test its effectiveness with other American Indian and Alaska Native tribes. However, this study will be the first to report on the effectiveness of a culturally tailored ACP intervention with an American Indian tribe. Our study results will provide valuable information for the health care community about American Indian readiness and confidence to engage in culturally tailored ACP. If more American Indians and Alaska Natives can express their health care wishes related to the end of life, it could lead to decreased suffering and hospitalization, increased hospice use, goal-concordant care, and increased patient and family satisfaction [6].

### Potential Problem and Alternate Plans

Recruitment for cohort 2 proved challenging. Many participants were unwilling to wait approximately 6 months to receive the intervention. This challenge led to a large difference in the number of participants enrolled in cohort 1 compared to cohort 2. Our planned analyses should determine if there are any differences between the cohorts, however, which will account for this discrepancy.

## Abbreviations

ACP: advance care planning
CAB: community advisory board
CBPR: community-based participatory research
MY WAY: Make Your Wishes About You
PAB: professional advisory board
POLST: Physician Orders for Life Sustaining Treatment
RCT: randomized controlled trial

## Data Availability

The data sets generated or analyzed during this study are not publicly available due to tribal institutional review board restrictions. Readers interested in accessing the data can contact the corresponding author on how to initiate tribal institutional review board permission.
